# Surviving the storm: A pragmatic non‐randomised examination of a brief intervention for disaster‐affected health and social care providers

**DOI:** 10.1111/hsc.14059

**Published:** 2022-10-05

**Authors:** Tara Powell, Jennifer Scott, Paula Yuma, Yuan Hsiao

**Affiliations:** ^1^ University of Illinois School of Social Work Urbana Illinois USA; ^2^ Louisiana State University, School of Social Work Baton Rouge Louisiana USA; ^3^ Colorado State University, School of Social Work Fort Collins Colorado USA; ^4^ Department of Communication University of Washington Seattle Washington USA

**Keywords:** disaster, health and social care providers, intervention, mental health

## Abstract

Disasters affect the well‐being of individuals, families and communities. Health and social care providers are essential in response and recovery efforts and are among the most vulnerable to negative physical and mental health impacts of a disaster. Few evidence‐based interventions are available to address the psychological needs of providers. The aim of this study was to examine the psychological distress of health and social care providers before and after participating in the brief group intervention, Resilience and Coping for the Healthcare Community (RCHC) and its expanded version, RCHC+. We conducted a pragmatic non‐randomised cluster trial with 762 health and social care providers in south Texas and Puerto Rico post‐Hurricanes Harvey and Maria. Participants completed surveys assessing post‐traumatic stress (PTSD), anxiety, burnout and secondary traumatic stress (STS) prior to intervention delivery and at two time points post‐intervention. We calculated the frequency of symptom cut‐off scores at baseline, then estimated multilevel ordinal models to examine changes in symptoms across time. Prior to participation in the RCHC (approximately 12 months after the hurricanes), providers reported high levels of PTSD, anxiety and STS symptoms. After participation, providers in both intervention conditions reported a significant reduction in PTSD symptoms from baseline that was sustained over both time points. The likelihood of a reduction in symptoms of anxiety and STS from baseline was sustained at both time points for participants in the RCHC+ condition. These findings indicate that both the RCHC and RCHC+ interventions may reduce psychological distress for health and social care providers and could be an important part of advance planning to support provider's mental health during and after a disaster. Further examination of the RCHC in other disaster contexts could provide additional insight into the responsiveness of the intervention to reducing psychological distress symptoms.

**What is known about this topic?**

- Health and social care providers are essential in disaster response and recovery efforts
- Health and social care providers are highly vulnerable to disaster‐related psychological distress
- There is a lack of evidence‐based interventions to support the well‐being of health and social care providers in post‐disaster contexts

**What this paper adds**

- The Resilience and Coping for the Healthcare Community (RCHC) and expanded RCHC+ reduced anxiety, secondary traumatic stress and post‐traumatic stress symptoms among hurricane‐affected health and social care providers
- Interventions such as the RCHC may support provider's mental health during the disaster recovery and reduce and/or mitigate psychological distress

## 1 INTRODUCTION

The consequences of environmental, technological and manmade disasters are both physical and psychological, and often have a lasting impact on survivors. Material damages to homes and communities, as well as injury and interpersonal loss, lead to multifaceted hardships (Shultz & Galea, 2017). It can take months and even years to recover, leading to sustained chronic stress for survivors (Johannesson et al., 2015; Nillni et al., 2013). Frontline health and social care providers are among the most vulnerable during and after a disaster as they are tasked with navigating limited resources and increased demand for medical care, counselling and assistance (Brooks et al., 2019; Krystal, 2020). These providers deliver essential services to survivors; however, their psychological needs are often overshadowed by the needs of their patients and clients putting them at high risk for psychological distress (Benedek et al., 2007; Powell et al., 2022).

Few evidence‐based interventions are available to reduce distress and address the unique circumstances of health and social care providers in post‐disaster contexts (Bercier & Maynard, 2015; Pollock et al., 2020). To better understand how to attend to the psychological needs of health and social care providers in post‐disaster settings, we conducted a pragmatic non‐randomised cluster trial examining the psychological distress of health and social care providers before and after participating in a brief group intervention, Resilience and Coping for the Healthcare Community (RCHC), delivered in the greater Houston area post‐Hurricane Harvey and in Puerto Rico post‐Hurricane Maria. We examined how participation in RCHC or its expanded version, RCHC+, affected the trajectory of post‐traumatic stress (PTSD), anxiety, burnout and secondary traumatic stress (STS) among providers.

### 1.1 Health and social care providers post‐disaster mental health

When a disaster strikes, health and social care providers step to the frontline to deliver immediate physical and mental health assistance, as well as link survivors to other essential services (Chandra & Acosta, 2010; Putra & Petpichetchian, 2011; Rowlands, 2013). Throughout recovery, they provide ongoing care to support individuals, families and communities in their efforts to regain a sense of normalcy (Chandra & Acosta, 2010; Pyles, 2007; Rowlands, 2013).

Many health and social care providers are also residents in disaster‐affected communities and therefore process their own trauma in tandem with that of their patients and co‐workers (Cocker & Joss, 2016). One systematic review found that disaster response significantly adversely impacted healthcare providers' psychological well‐being, resulting in PTSD, depression, substance abuse, anxiety disorders, somatic complaints and sleep disruption (Naushad et al., 2019). Clinical levels of PTSD have been found in 16% to over 40% of providers post‐disaster (Brooks et al., 2019; Naushad et al., 2019; Powell et al., 2022), a rate significantly higher than the approximately 10% for the general public (Beaglehole et al., 2018).

Conditions such as secondary traumatic stress (STS) and burnout also disproportionately affect disaster‐affected health and social care providers (Brooks et al., 2019). STS mirrors PTSD symptomology, but surfaces after listening to experiences of trauma rather than experiencing trauma directly (Bride, Robinson, Yegidis, & Figley, 2004). STS is common among frontline providers as they frequently interact with trauma survivors (Kanno & Giddings, 2017; Von Rueden et al., 2010). One study found approximately 70% of providers reported high levels of STS symptoms after Superstorm Sandy struck the north‐eastern United States in 2013 (Moses, 2015). During the COVID‐19 pandemic, STS has been detected in over 50% of health and social care workers (Holmes et al., 2021; Orrù et al., 2021a). Symptoms of burnout externalise in work‐related behaviours, including disengagement, cynicism and/or inefficacy in tasks (Aronsson et al., 2017), result from exhaustion from performing emotionally demanding jobs (Cieslak et al., 2014; Mateen & Dorji, 2009). Burnout has become especially prevalent among healthcare providers in the wake of the COVID‐19 pandemic disaster – of over 3500 healthcare providers screened in three counties in 2020, 67% screened at high risk for burnout (Denning et al., 2021).

Factors attributed to psychological distress among health and social care providers post‐disaster include the degree of exposure (e.g. deceased or missing relatives and near‐death experiences), pre‐existing psychopathology, work‐related conditions (e.g. amount of rest, communication and training), demographic factors (i.e. age and gender) and degree of social support (Carmassi et al., 2020; Guilaran et al., 2018; Jalili et al., 2021; Wu et al., 2009). Additionally, post‐disaster psychological distress may be attributable to providers' heightened vulnerability to occupational stress given the nature of their day‐to‐day work (Esterwood & Saeed, 2020; Goh & Agius, 2010).

### 1.2 Interventions for health and social care providers

Psychological distress impacts providers' ability to deliver essential services and be retained in these specialised fields (Kanno & Giddings, 2017; Labrague & Ballad, 2021; Tabur et al., 2022). This, alongside the unparalleled increase in environmental, biological and man‐made disasters, underscores the need for interventions to support their psychological health (Søvold et al., 2021). Although some interventions have been developed, evidence‐based or evidence‐informed interventions to address their psychological needs are limited (Bercier & Maynard, 2015; Cocker & Joss, 2016). One systematic review of interventions designed to address STS among providers found no rigorously tested interventions that were effective in reducing secondary trauma (Bercier & Maynard, 2015). Another recent Cochrane review found limited evidence base for interventions specifically designed to support the psychological well‐being of health and social care workers during a pandemic disaster (Pollock et al., 2020).

One manualised intervention designed to address the specific needs of health and social care providers in the aftermath of a disaster, Resilience and Coping for the Healthcare Community (RCHC), was originally developed and tested in Federally Qualified Health Centers (FQHCs) impacted by Hurricane Sandy in the states of New York and New Jersey, United States (Powell & Yuma‐Guerrero, 2016; Yuma et al., 2019). A mixed‐methods evaluation of that delivery found significant positive benefits in terms of knowledge and social support from baseline to 3‐week follow‐up (Powell & Yuma‐Guerrero, 2016). To better understand how the RCHC addresses psychological needs, we examined how participation in the intervention affected the trajectory of post‐traumatic stress (PTSD), anxiety, burnout and secondary traumatic stress (STS) among frontline providers in the aftermath of two major hurricanes that struck Texas and Puerto Rico in 2017. Additionally, we compared the effects of participation in the RCHC to participation in an expanded version, RCHC+.

## 2 MATERIALS AND METHODS

We conducted a pragmatic non‐randomised cluster trial in two post‐disaster contexts, South Texas post‐Hurricane Harvey and Puerto Rico post‐Hurricane Maria. The pragmatic design enabled assessment in ‘real world’ practice settings (Patsopoulos, 2022). We analysed the odds of experiencing an improvement in psychological distress symptoms in terms of four measures (anxiety, PTSD, burnout and STS) at two time points after participating in either the RCHC or RCHC+: post‐intervention (12‐ = weeks post‐baseline) and follow‐up (18 weeks post‐baseline).

### 2.1 Context

In the summer 2017, three major hurricanes struck the United States, causing widespread damage and destruction. On August 25, Hurricane Harvey made landfall as a Category 4 Hurricane. Harvey stalled over Southeast Texas for days, dropping more than 33 trillion gallons of rain‐producing massive flooding in Houston, Beaumont, and the surrounding communities (Van Oldenborgh et al., 2017). More than 185,000 homes were damaged or destroyed, approximately 42,000 people were displaced to shelters, and 364,000 registered for FEMA assistance (Federal Emergency Management Agency, 2017). Shortly thereafter, Hurricanes Irma and Maria struck the island of Puerto Rico in quick succession, causing catastrophic devastation. The electrical grid failed across the entire island, many were left without water service and thousands were displaced from their homes (Zorrilla, 2017). A study conducted by the Puerto Rican government and public health researchers estimated 2975 died as a result of the storm (Santos‐Burgoa et al., 2018). Although damage was widespread in both areas, aid was much slower to arrive in Puerto Rico where over a million residents remained without power 6 months after Maria (Einbinder, 2018; Kwasinski et al., 2019; Lichtveld, 2018; Scaramutti et al., 2019).

### 2.2 Resilience and coping for the healthcare community intervention (RCHC)

**FIGURE 1:**
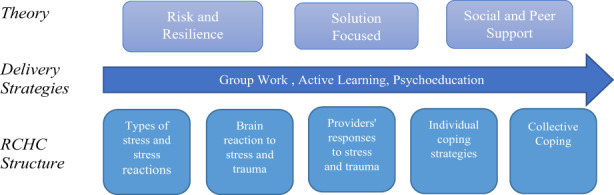
RCHC conceptual model

The RCHC is a brief, group intervention designed to address the specific needs of disaster survivors who serve as frontline health and social care providers in the aftermath of a disaster (Powell & Yuma‐Guerrero, 2016; Yuma et al., 2019). The aims of RCHC are threefold: (i) enhance knowledge of stress, (ii) reduce stress and distress and (iii) increase healthy coping strategies. The intervention design integrates action learning (Waddill & Marquardt, 2016) and solution‐focused (Sharry, 2007) techniques to support participants in the identification of both individual and collective healthy coping strategies and social supports. As depicted in Figure 1, RCHC brings together three theories of intervention: (1) risk and resilience (Greene, 2017), (2) solution‐focused support (Sharry, 2007) and (3) social and peer support (Lakey, 2000). It is delivered in a psychoeducational group formatted around five structured themes. RCHC is delivered by a pair of facilitators, at least one of which is a licensed mental health professional, to small groups of 10–15 in one 3‐hour session, followed by a 1‐h booster session 2 to 4 weeks after participation in the initial intervention (see Yuma et al., 2019, for a detailed description of the intervention).

### 2.3 Resilience and coping for the healthcare community plus intervention (RCHC +)

The RCHC+ is an extension of the RCHC intervention that adds two additional 1‐hour sessions in the 3 months following the initial 3‐h RCHC session. These sessions include one psycho‐educational training and one wellness group. The psycho‐educational training session focused on providing basic support and referrals to individuals who may be currently experiencing, or at risk of, psychological distress. The wellness group included a 1‐hour session focused on reinforcing the healthy coping skills discussed in RCHC and managing on‐going post‐disaster stressors (see Yuma et al., 2019, for a detailed description of the intervention).

### 2.4 Participants and procedures

We recruited a total of 38 health and social service agencies in Puerto Rico (*n* = 24) and Texas (*n* = 14) that agreed to offer their employees the opportunity to participate in the intervention and study between June and August of 2018. Agencies included FQHCs, hospitals, disaster‐ = response agencies and community‐based health and behavioural health centres identified by research study coordinators from lists of service providers operating during the immediate post‐disaster period in partnership with AmeriCares, the international humanitarian disaster response organisation that funded and provided staff to conduct this intervention and study. Agency contacts also referred other agencies. Agencies were recruited from across each location (i.e. the entire island of Puerto Rico, and the Houston and Corpus Christi Metropolitan Service Areas).

To recruit agencies, research coordinators approached organisational leaders to offer the intervention and provide information about study participation. Agencies that agreed to participate chose which condition (RCHC or RCHC+) based on their facility's needs and availability. The rationale provided by agency personnel regarding their choice to research coordinators focused on logistical constraints. This pragmatic design was developed as a direct result of feedback from early agency partners who made it clear that random assignment to an intervention condition would not be feasible for their agencies.

After an agency consented to participate, research coordinators worked with agency administrators to schedule the intervention session(s) in accordance with their assigned study condition (RCHC and RCHC+). To recruit participants within the organisation, information was provided through fliers and emails about RCHC, the study and time it would be delivered. Providers were eligible if they were as follows: (1) over 18 years of age; (2) able to speak and read English or Spanish; (3) experienced Hurricane Harvey or Maria and (4) were currently employed and working for a participating partner agency currently and at the time of the Hurricane. In collaboration with agency administrators, the research coordinators obtained a list of individuals who expressed interest and signed up to take part in the intervention.

In Puerto Rico, all agencies in both RCHC and RCHC+ intervention groups chose to conduct the intervention and accompanying assessments in Spanish. Americares contracted the initial translation of the intervention curriculum which was then revised to fit the nuances of Puerto Rican Spanish by the programme coordinators, native Puerto Ricans. The full structure and all the psychoeducational content of the intervention remained the same. One activity that served to engage participants prior to later content delivery was modified to better fit the post‐Maria Puerto Rican context; however, neither the structure nor purpose of the activity was changed.

Participants received either RCHC or RCHC+ and completed scales that assessed indicators of anxiety, PTSD, burnout and STS at baseline and two follow‐up time points: post‐intervention (12 weeks post‐baseline) and follow‐up (18 weeks post‐baseline). Approximately 1 week prior to the intervention, those who expressed interest were sent an electronic link to the informed consent form and baseline assessment to complete prior to receipt of programme services; agencies also had the option of using paper forms for participants. Participants were also given the option to consent and complete the baseline assessment at the time of the initial RCHC session. All participants were provided a $10 gift card incentive for completion of each survey. The study was approved by the Institutional Ethical Review Board at the University of Illinois Urbana, Champaign.

**FIGURE 2:**
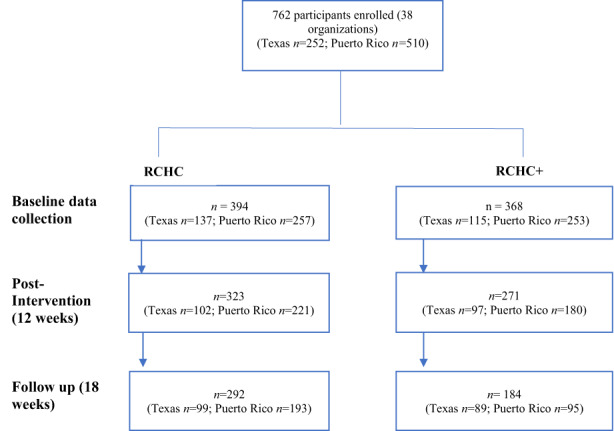
Flowchart of recruitment and retention of participants throughout the study

Participants included health and social care providers (e.g. doctors, nurses, social workers and case managers). A total of 762 participants (between 10 and 20 at each agency) consented to participate in the study (RCHC *n* = 394; RCHC+ *n* = 368) and completed the baseline assessment. Retention across the study time points is shown in Figure 2. After baseline data collection, and prior to intervention delivery, four agencies with *n* = 52 participants dropped out of the study (*n* = 21 RCHC, *n* = 30 RCHC+), citing time and resource constraints. At post‐intervention (12 weeks), 694 of the 710 participants remained in the study and completed assessments. At follow‐up (18 weeks), 476 participants were retained. This manuscript presents the results of both the 12‐ and 18‐week measures and therefore, the data the 476 individuals retained for the entire study period were analysed and presented here.

The majority of participants were female (79%), married (56.2%) and college educated, with nearly 60% having a bachelor's degree (30%) or a master's degree or higher (27.7%) (Table 1). About half of the participants experienced damage to their own homes during the hurricanes, this experience was significantly more likely for the participants in Puerto Rico. Otherwise, participants in Puerto Rico and Texas were relatively similar in demographics, as were participants across RCHC and RCHC+ study conditions.

**TABLE 1:** Participant characteristics (*N* = 476)

	Puerto Rico *n* (%)		Texas *n* (%)		Total *N* (%)	Chi square
	RCHC	RCHC+	RCHC	RCHC+		
Gender						
Male	32 (16.6)	24 (25.5)	19 (19.2)	20 (22.5)	95 (20.0)	3.60
Female	160 (82.9)	70 (74.5)	80 (80.8)	69 (77.5)	379 (79.8)	
Age						
18–25	9 (4.9)	4 (4.7)	13 (13.1)	6 (6.7)	32 (7.0)	
26–34	40 (22.0)	27 (31.8)	33 (33.3)	22 (24.7)	122 (26.8)	6.21
35–44	50 (27.5)	26 (30.6)	21 (21.2)	22 (24.7)	119 (26.2)	
45–54	55 (30.2)	19 (22.4)	16 (16.2)	18 (20.2)	108 (23.7)	
55+	28 (15.3)	9 (10.6)	16 (16.2)	21 (23.6)	74 (16.3)	
Marital status						
Single	59 (31.2)	22 (23.7)	35 (35.4)	34 (38.2)	150 (31.9)	
Married/living with partner	103 (54.5)	64 (68.8)	51 (51.5)	46 (51.7)	264 (56.2)	5.43
Divorced	21 (11.1)	3 (3.2)	11 (11.1)	9 (10.1)	44 (9.4)	
Widowed	2 (1.1)	1 (1.1)	1 (1.0)	0 (0.0)	4 (0.9)	
Other	4 (2.2)	3 (3.2)	1 (1.0)	0 (0.0)	2 (0.4)	
Education						
High school	1 (0.5)	0 (0.0)	16 (16.2)	11 (12.4)	28 (5.9)	6.42
Some college	9 (4.7)	5 (5.4)	31 (31.3)	18 (20.2)	63 (13.3)	
Bachelors	57 (29.7)	20 (21.5)	29 (29.3)	36 (40.4)	142 (30.0)	
Masters or higher	76 (39.6)	25 (26.9)	12 (12.1)	18 (20.2)	131 (27.7)	
Other (e.g. nursing school, medical school)	49 (25.5)	43 (46.2)	11(11.0)	6 (6.6)	92 (19.5)	
Home damage						
Yes	116 (60.1)	53 (56.4)	43 (43.4)	30 (33.7)	242 (50.9%)	3.72*
No	77 (39.9)	41 (43.6)	56 (56.6)	59 (66.3)	233 (49.1%)	
Total sample	193 (66.1)	95 (51.6)	99 (33.9)	89 (48.4)	476	9.88^**^

### 2.5 Measures

Participants responded to a baseline questionnaire between 10 and 12 months after the hurricanes and follow‐up questionnaires at two additional time points, 12‐ and 18 weeks post‐intervention. At baseline, the questionnaire collected general demographic information (e.g. age, gender and education) and the impact of the hurricane (e.g. home damage). Participants' well‐being was assessed at all three time points using three standardised measures: the Impact of Events Scale Revised (IES‐R) for PTSD symptoms (Beck et al., 2008), the Beck Anxiety Inventory (BAI) for anxiety symptoms (Beck et al., 1993), and the Professional Quality of Life scale (ProQOL) for burnout and STS (Stamm, 2010). Each measure assessed is described as follows.

#### 2.5.1 PTSD

The IES‐R is a 22‐item self‐report measure of post‐traumatic stress symptoms. Respondents are asked to identify a specific stressful event and indicate their level of distress during the past 7 days. Items range from 0 (not at all) to 4 (extremely). Questions measured clusters of PTS, including avoidance (e.g. I stayed away from reminders about it), intrusion (e.g. Any reminder brought back feelings about it) and hyperarousal (e.g. I was jumpy and easily startled). IES‐R scores range from 0 to 88; a score of 0–23 is representative of low PTSD symptoms and 24–32 indicate that partial PTSD symptoms are present and of clinical concern. A score of 33–38 represents the clinical cut‐off for a probable diagnosis of PTSD, and a score of 39 or more indicates symptoms severe enough to suppress one's immune system functioning (Beck et al., 2008; Creamer et al., 2003). The reliability of the IES‐R in our sample was high at α = 0.94.

#### 2.5.2 Anxiety

The Beck Anxiety Inventory (BAI) was used to measure clinical anxiety symptoms in adults (Beck et al., 1988, 1993). A 21‐item index of Likert questions assessed the severity of symptoms in the past month from 0 (not at all) to 3 (severe – it bothered me a lot). Items included symptoms such as ‘feeling nervous’, ‘feeling shaky or unsteady’ and ‘fear of losing control’. Summed BAI scores range from 0 to 63, minimal anxiety is indicated by scores from 0 to 7, mild anxiety from 8 to 15, moderate anxiety from 16 to 25 and severe anxiety from 30 to 63. The reliability of the BAI in our sample was α = 0.93.

#### 2.5.3 Burnout and STS

The Professional Quality of Life (ProQOL‐5) scale, one of the most widely used scales to measure the professional quality of life among healthcare professionals (Stamm, 2010), measured burnout (work‐related frustration, hopelessness, and exhaustion) and STS (distress related to hearing of others trauma). The ProQoL‐5 consists of 30 items scored on a 5‐point Likert scale (1 = never to 5 = very often). The burnout subscale included 10 items with scores ranging from 1 to 50 (e.g. ‘I feel trapped by my job’ and ‘I feel worn out because of my work’) (Stamm, 2010). Cut‐off scores for burnout included: low (19 or less), medium (20 to 23) and high (27 or above). The STS subscale included 10 items with scores ranging from 1 to 50 (e.g. ‘I am not as productive at work because I am losing sleep over traumatic experiences of a person I help at work’, and ‘I feel depressed because of the traumatic experiences of people I help’). STS cut‐off scores included: low (13 or less), middle (14 to 17) and high (above 18; De La Rosa, 2018). The reliability of burnout in our sample was α = 0.72, and STS was α = 0.77.

### 2.6 Analyses

We first calculated the frequency, stratified by location (Puerto Rico, Texas) and study condition (RCHC, RCHC+), of symptom cut‐off scores at baseline for the mental health measures (e.g. low, middle and high). We then estimated multilevel ordinal models to examine the association between participation in the RCHC or RCHC+ and change in PTSD, anxiety, burnout and STS symptoms across time. We analysed improvement in outcomes by calculating the odds of movement from a higher to a lower severity category at the 12‐ and 18‐week measurement timepoint, using ordinal mental health outcome variables (1 = change and 0 = no change) as they provide a better mechanism for analysing clinical significance of intervention benefits (Hedeker, 2015). Such ordinal models allow us to assess if respondents move from a category of higher clinical concern to a less concerning category. The multilevel ordinal model reflected two levels: (Level 2) symptoms at time points (i.e. 12 and 18 weeks) nested within (Level 1) individuals. We estimated the following cumulative logit link mixed model for week 12:

$$g\left(Y_{\mathrm{it}}\right)=\beta_{0}+\beta_{1}X_{\mathrm{RCH}C_{W12}}+\beta_{2}X_{\mathrm{RCHC}{+}_{W12}}+\alpha_{i}+\varepsilon_{\mathrm{it}}$$

$g\left(Y_{\mathrm{it}}\right)$ represents the ordinal logit transformation of the outcome (positive change in mental health condition) for participant *i* at time *t*. *RCHC* represents the RCHC intervention, and *RCHC+* represents the RCHC plus intervention. $X_{\text{RCHC}\_W12}$ and $X_{\text{RCHC}+\_W12}$ indicate which intervention the participant received (RCHC or RCHC+) at post‐assessment (*W12*). $\beta_{1}$ and $\beta_{2}$ represent the intervention effects. The participant‐level random intercept $\alpha_{i}$ accounts for the correlated observations for the same participant. We estimated the following cumulative logit link mixed model for week 18:

$$g\left(Y_{\mathrm{it}}\right)=\beta_{0}+\gamma_{1}X_{\mathrm{RCHC}\_W18}+\gamma_{2}X_{\mathrm{RCHC}+\_W18}+\alpha_{i}+\varepsilon_{\mathrm{it}}$$

We estimated an additional model for each outcome including controls for gender, age and location (Puerto Rico and Texas). As the models with controls produced similar results (available upon request), we report results without controls to maximise power. Coefficients are the log‐odds of increasing from a lower to a higher degree of mental health symptoms (e.g. from low to medium PTSD). Positive coefficients indicate participants are more likely to have increased in level of symptomatology; negative coefficients indicate participants are more likely to have decreased their level of symptoms. We used SPSS statistical software (version 22.0) to estimate descriptive statistics and *R* (version 3.5.1) and the *ordinal* package (Christensen, 2019) to estimate the multilevel ordinal models.

## 3 RESULTS

At baseline, 19.1% of participants reported symptoms above the clinical cut‐off for a probable diagnosis or severe PTSD, slightly more (21.7%) in RCHC than in RCHC+ (19.2%) condition. Participants from Puerto Rico reported severe levels of PTSD at a higher rate (20%) than individuals from Texas (7%). Approximately 18.9% of all participants (22.6% RCHC; 17.6% RCHC+) reported moderate‐to‐severe anxiety. High rates of STS were also observed in both conditions prior to participation; 50.7% of participants in RCHC and 45.9% of participants in the RCHC+ condition reported symptoms in the high range for STS. Rates of burnout were lower, with 8.6% of participants in the RCHC and 6.6% of participants in RCHC+ reporting symptoms in the high range. Table 2 presents participant mental health symptoms at baseline stratified by study condition and geographic location.

**TABLE 2:** Mental health symptoms at baseline by intervention condition and location (*n* = 476)

Symptom	RCHC *n* (%)			RCHC+ *n* (%)			Chi square
	Texas	PR	Total	Texas	PR	Total	
Post‐traumatic stress							
Low	77 (81.9)	81 (46.6)	158 (59.0)	60 (73.2)	55 (61.1)	115 (66.9)	3.42
Clinical Concern	8 (8.5)	44 (25.3)	52 (19.4)	13 (15.9)	11 (12.2)	24 (14.0)	
Clinical Cut‐off	1 (1.1)	12 (6.9)	13 (4.9)	4 (4.9)	5 (5.6)	9 (5.2)	
Severe	8 (8.5)	37 (21.3)	45 (16.8)	5 (6.1)	19 (21.1)	24 (14.0)	
Anxiety							
Minimal	65 (73.0)	96 (54.2)	161 (60.5)	58 (60.7)	54 (60.7)	112 (65.5)	4.72
Mild	10 (11.2)	35 (19.8)	45 (16.9)	15 (18.3)	14 (15.7)	29 (17.0)	
Moderate	9 (10.1)	30 (16.9)	39 (14.7)	5 (6.1)	9 (10.1)	14 (8.2)	
Severe	5 (1.9)	16 (9.0)	21 (7.9)	4 (4.9)	12 (13.5)	16 (9.4)	
Secondary traumatic stress							
Low	12 (6.2)	8 (8.1)	20 (6.8)	6 (6.7)	4 (4.3)	10 (5.5)	1.82
Medium	76 (39.4)	48 (48.5)	124 (42.5)	35 (39.3)	54 (57.4)	89 (48.6)	
High	105 (54.4)	43 (43.4)	148 (50.7)	48 (53.9)	36 (38.3)	84 (45.9)	
Burnout							
Low	44 (44.4)	88 (45.6)	132 (45.2)	49 (55.1)	52 (55.3)	101 (55.2)	4.52
Medium	42 (42.4)	93 (48.2)	135 (46.2)	35 (39.3)	35 (37.2)	70 (38.3)	
High	12 (4.5)	12 (6.2)	25 (8.6)	5 (5.6)	7 (7.4)	12 (6.6)	

We found that participants in both the RCHC and RCHC+ conditions had a higher likelihood of reporting a reduction in PTSD symptoms from baseline at both time points post‐intervention. At week 12, participants who received RCHC were 73% more likely to score in a lower severity category on PTSD symptoms from baseline (log‐odds −1.30). At week 18, participants remained 72% more likely to score in a lower severity category on PTSD symptoms from baseline (log‐odds −1.26). Similarly, participants in RCHC+ were significantly more likely to move from a higher to lower PTSD category from baseline at both time points (72% and 73%, respectively), as seen in Table 3.

**TABLE 3:** Log‐odds change in mental health symptoms from baseline by intervention condition

Condition	Changes in mental health symptoms, log‐odds [95% CI]							
	Anxiety		Post‐traumatic stress		Burnout		Secondary traumatic stress	
	W12	W18	W12	W18	W12	W18	W12	W18
RCHC	−0.72* [−1.53,0.09]	−0.64 [−1.30,0.01]	−1.30** [−2.18, −0.42]	−1.26*** [−1.97, −0.55]	−0.49 [−0.18, 1.15]	0.40 [−0.12, 0.93]	−0.70* [−1.33, −0.07]	−0.51 [−1.02, 0.01]
RCHC+	−0.80** [−1.38, −0.23]	−0.69* [−1.24, −0.13]	−1.29*** [−1.91, −0.67]	−1.32*** [−1.89, −0.74]	−0.07 [−0.55, 0.41]	−0.09 [−0.52, 0.34]	−0.86*** [−1.31, −0.41]	−0.77*** [−1.17, −0.37]
N obs	638	700	659	725	709	768	710	766
N subjects	423	430	425	435	443	443	443	443

*Note*: Results are reported as odds ratios, and confidence intervals in parentheses.

*Note*: The coefficients of the treatment effects represent the change in the outcome compared to the baseline. Since the model includes fixed effects for participants, the effects are within‐individual differences.

*** *p* < 0.001

** *p* < 0.01

* *p* < 0.05.

Levels of anxiety also decreased for participants in both intervention conditions, although the reduction was not significant for those in the RCHC group at the first post‐intervention assessment (12 weeks). For participants in the RCHC+ condition, the log‐odds of moving from a higher to lower anxiety category were − 0.80 (equivalent to odds of 55%) at week 12. By week 18, the likelihood of a reduction in anxiety from baseline for both conditions was significant, 50% for participants in the RCHC+ group (log‐odds −0.69) and 47% for participants in the RCHC group (log‐odds −0.64). As shown in Table 3, both groups experienced a roughly equivalent and significant likelihood of experiencing a reduction in anxiety from baseline at week 18; however, the RCHC+ group retained a slightly steeper difference in the likelihood of symptom reduction.

Participants in both RCHC and RCHC+ were also significantly more likely to experience a reduction in STS from baseline at week 12. The magnitude of change was smaller (log‐odds of −0.70) for the RCHC group than for the RCHC+ group (log‐odds of −0.86). At week 18, the likelihood of a reduction in STS from baseline was only significant for the RCHC+ group, although the magnitude of the likelihood of reduction had decreased (log odds of −0.77). As shown in Table 3, neither the RCHC nor RCHC+ groups showed a significant reduction in burnout from baseline at either time point.

## 4 DISCUSSION

The present study examined the effects of the RCHC and expanded RCHC+ on health and social care providers' mental health in two hurricane‐affected regions. We found high rates of psychological distress among health and social care providers in both Texas and Puerto Rico after experiencing major Hurricanes in 2018, and evidence that participation in RCHC and expanded RCHC+ may positively impact providers' mental health. Prior to participation in RCHC (approximately 12 months after the hurricanes), providers reported high levels of PTSD, anxiety and STS symptoms. After participation, we found that providers reported significant reductions in mental health symptoms across outcomes in both intervention conditions. The likelihood of a reduction in symptoms of anxiety and STS from baseline was sustained at both time points for participants in the RCHC+ condition.

At baseline (approximately 12 months after the hurricanes), providers in our study reported higher PTSD symptoms than reported in the general population (19.2% above the clinical threshold as compared to 10%; Beaglehole et al., 2018). Participants from Puerto Rico reported severe PTSD at a higher rate (~20%) than those in Texas (~7%). Similarly, a larger proportion of providers in Puerto Rico reported moderate‐to‐severe anxiety than providers in Texas. This difference may be attributed, at least in part, to the wide disparities in aid for recovery between the two locations. Although both Hurricanes Harvey and Maria caused widespread damage and destruction, aid was much slower to arrive in Puerto Rico (Einbinder, 2018; Lichtveld, 2018). After Hurricane Harvey power was restored within weeks, whereas in Puerto Rico over a million residents remained without power and many homes were still uninhabitable 6 months after Maria (Kwasinski et al., 2019; Scaramutti et al., 2019).

We also found a high rate of STS among providers overall at baseline (45.9%); a rate consistent with studies of STS among providers responding to environmental (Bauwens & Tosone, 2014), pandemic (Holmes et al., 2021; Orrù et al., 2021b) and man‐made disasters (Ebren et al., 2022). Although the baseline data were obtained almost 1 year after the hurricanes, the communities in our study were still recovering and health and social care workers still providing ongoing and continuous care to survivors. These high rates of mental health distress among providers prior to the implementation of RCHC reinforce the appropriateness of intervention to support health and social care providers, even when delivered well into the disaster recovery.

With regards to post‐intervention outcomes, we found a significant likelihood of experiencing a reduction in PTSD from baseline for both intervention groups at 12 weeks post‐intervention that was sustained through the 18‐week follow‐up assessment. Although we can also not account for how much of the improvement in symptoms is due to natural recovery over time as some decrease in PTSD symptoms is expected in longitudinal studies (Cukor et al., 2011; Hu et al., 2016), the reduction in PTSD from baseline seen at 18 week follow‐up for both intervention groups was level with 12 weeks post‐intervention, during which time there were no intervention sessions. This pattern suggests that symptom improvement could be attributed, at least in part, to participation in the intervention.

Participants in the RCHC+ cohort also reported a significant likelihood of a reduction in anxiety and STS from baseline that was sustained between post‐intervention and follow‐up, however, we did not observe the same trajectory in RCHC participants. A significant likelihood of a decrease in STS was only observed post‐intervention (12 weeks), and of anxiety only at the 18‐week follow‐up for those in the RCHC cohort. Taken together, however, we suspect that the lack of a significant difference in the changes among individuals who participated in the RCHC may be due to the larger standard errors resulting from a smaller sample size rather than a true non‐effect at the population level. Although these findings appear to suggest the more intensive RCHC+ intervention may yield greater benefits for providers in post‐disaster settings; further examination with larger sample sizes is needed to substantiate this distinction.

Participants in both intervention conditions were slightly more likely to report a reduction in burnout from baseline, however, not to a significant degree. There are several potential reasons for this finding. First, high levels of burnout were not detected prior to participation in either intervention, the majority of participants (55.2%) reported low levels. Therefore, it was unlikely we would observe a significant reduction in burnout given the limited opportunity for improvement (Hedeker, 2015). Additionally, both intervention conditions focus on the micro (individual) and mezzo (group) levels, but did not address the macro (organisational) factors such as increased workloads and low institutional support that often contribute to burnout (Galanis et al., 2021; Mattei et al., 2017). In situations like the current COVID‐19 pandemic where burnout is of significant concern, consideration could be given to adapting the RCHC to support practitioners in addressing concerns through organisational policy.

Our study yielded promising findings; however, several limitations should be noted. First, our real‐world post‐disaster services context did not allow for a controlled trial due to the continuously changing context of the disaster response organisations, and the threat of additional weather events in the year following Harvey and Maria. Discussions with partnering agency stakeholders early in the design process identified these challenges, which we addressed by using a pragmatic design. Pragmatic designs may produce outcomes more readily generalisable to real‐life practice settings than highly controlled trials (Ford & Norrie, 2016; Glasgow, 2013). However, this prevented us from enlisting true control sites and necessitated providing participating agencies the choice regarding participation in the RCHC or RCHC+ intervention condition. As we cannot account for alternative explanations for the improvements in outcomes without a control condition, our design does not meet the criteria for internal validity (Glasgow, 2013). Given the nature of the post‐disaster context and our partner agencies' challenges, we prioritised the agency's needs despite the limitations to our design.

## 5 CONCLUSION

High levels of mental health distress persisted among health and social care providers in Texas and Puerto Rico nearly a year after the hurricanes, and providers experienced a significant and sustained reduction in mental health symptoms (PTSD, anxiety and STS) after participation in the RCHC and RCHC+ intervention. These findings have occupational health implications for health and social care providers who work in disaster response and recovery efforts in the United States and internationally. Given the high levels of psychological distress among providers during response and recovery, employers and disaster recovery organisations could better anticipate the need for mental health support and incorporate strategies to mitigate symptoms. Advanced planning for employee care and evaluating policies to minimise shift burden during response and recovery could reduce distress and improve the resiliency of the workforce.

Additionally, we found that both the RCHC and RCHC+ interventions may reduce psychological distress, suggesting they could be an important part of advance planning to support provider's mental health during a disaster. Incorporating the intervention into organisational scheduling during the response and recovery period could support providers in mitigating distress. More research is needed, however, to clarify the effectiveness of RCHC. Studies that include control groups, as well as the examination of the RCHC with other populations, such as individuals who are not first responders and members of other occupations, such as teachers, clergy and law enforcement officers, across a wider variety of disaster contexts both in the United States and internationally could add to the assessment of the intervention's efficacy. Additionally, further examination on how RCHC affects psychological distress in contexts of consistent and sustained disaster could provide insight into the responsiveness of RCHC to more enduring symptoms.

## AUTHOR CONTRIBUTIONS

Conception and design of work (TP, JS, PY and YS); data collection (TP, JS and PY); data analysis and interpretation (YS, JS, TP and PY) and manuscript preparation (TP, JS, PY and YS).

## CONFLICT OF INTEREST

The authors have no potential conflicts of interest to disclose.

## Data Availability

The data that support the findings of this study are available from the corresponding author, [TP], upon reasonable request.
